# Scale Drop Disease Virus (SDDV) and *Lates calcarifer* Herpes Virus (LCHV) Coinfection Downregulate Immune-Relevant Pathways and Cause Splenic and Kidney Necrosis in Barramundi Under Commercial Farming Conditions

**DOI:** 10.3389/fgene.2021.666897

**Published:** 2021-06-18

**Authors:** Jose A. Domingos, Xueyan Shen, Celestine Terence, Saengchan Senapin, Ha Thanh Dong, Marie R. Tan, Susan Gibson-Kueh, Dean R. Jerry

**Affiliations:** ^1^Tropical Futures Institute, James Cook University, Singapore, Singapore; ^2^Centre for Sustainable Tropical Fisheries and Aquaculture, James Cook University, Townsville, QLD, Australia; ^3^Faculty of Science, Fish Health Platform, Center of Excellence for Shrimp Molecular Biology and Biotechnology (Centex Shrimp), Mahidol University, Bangkok, Thailand; ^4^National Center for Genetic Engineering and Biotechnology (BIOTEC), National Science and Technology Development Agency (NSTDA), Pathumthani, Thailand; ^5^Faculty of Science and Technology, Suan Sunandha Rajabhat University, Bangkok, Thailand; ^6^School of Applied Science (SAS), Republic Polytechnic, Singapore, Singapore

**Keywords:** transcriptome (RNA-seq), *Lates calcarifer* herpes virus, immunity, disease outbreak, scale drop disease virus, Asian sea bass (*Lates calcarifer*)

## Abstract

Marine farming of barramundi (*Lates calcarifer*) in Southeast Asia is currently severely affected by viral diseases. To better understand the biological implications and gene expression response of barramundi in commercial farming conditions during a disease outbreak, the presence of pathogens, comparative RNAseq, and histopathology targeting multiple organs of clinically “sick” and “healthy” juveniles were investigated. Coinfection of scale drop disease virus (SDDV) and *L. calcarifer* herpes virus (LCHV) were detected in all sampled fish, with higher SDDV viral loads in sick than in healthy fish. Histopathology showed that livers in sick fish often had moderate to severe abnormal fat accumulation (hepatic lipidosis), whereas the predominant pathology in the kidneys shows moderate to severe inflammation and glomerular necrosis. The spleen was the most severely affected organ, with sick fish presenting severe multifocal and coalescing necrosis. Principal component analysis (PC1 and PC2) explained 70.3% of the observed variance and strongly associated the above histopathological findings with SDDV loads and with the sick phenotypes, supporting a primary diagnosis of the fish being impacted by scale drop disease (SDD). Extracted RNA from kidney and spleen of the sick fish were also severely degraded likely due to severe inflammation and tissue necrosis, indicating failure of these organs in advanced stages of SDD. RNAseq of sick vs. healthy barramundi identified 2,810 and 556 differentially expressed genes (DEGs) in the liver and muscle, respectively. Eleven significantly enriched pathways (e.g., phagosome, cytokine-cytokine-receptor interaction, ECM-receptor interaction, neuroactive ligand-receptor interaction, calcium signaling, MAPK, CAMs, etc.) and gene families (e.g., tool-like receptor, TNF, lectin, complement, interleukin, chemokine, MHC, B and T cells, CD molecules, etc.) relevant to homeostasis and innate and adaptive immunity were mostly downregulated in sick fish. These DEGs and pathways, also previously identified in *L. calcarifer* as general immune responses to other pathogens and environmental stressors, suggest a failure of the clinically sick fish to cope and overcome the systemic inflammatory responses and tissue degeneration caused by SDD.

## Introduction

Barramundi (*Lates calcarifer*), also known as Asian seabass, is an important tropical aquaculture food fish and a species that is receiving increased global attention due to its good attributes for aquaculture and consumer attractiveness (Jerry, 2013). As the barramundi industry grows and production intensifies, commercial farmers have seen the occurrence of several emerging viral and bacterial diseases that affects the survival of farmed stocks (Gibson-Kueh et al., 2012; de Groof et al., 2015; Dong et al., 2017a,b; Chang et al., 2018; Chen et al., 2019; Girisha et al., 2020). Emerging diseases are a particular threat to intensive barramundi aquaculture, where fish are directly exposed to the natural environment. In these open systems, it is impractical, due to the unavailability of efficacious commercial vaccines.

Scale drop disease virus (SDDV), a novel *Megalocytivirus* of the *Iridoviridae* family, has had devastating consequences on the production of barramundi in Southeast Asia, particularly during early juvenile phases (∼50–500 g) reared in sea cages. SDDV is often associated with mortalities ranging from 40 to 50% of cultured stock (Gibson-Kueh et al., 2012; de Groof et al., 2015; Senapin et al., 2019). While initial reports of the disease were from farms in Singapore (Gibson-Kueh et al., 2012; de Groof et al., 2015), SDDV has been reported in other Southeast Asian producing countries, such as Malaysia (Nurliyana et al., 2020), Thailand (Senapin et al., 2019), and Indonesia (de Groof et al., 2015). SDD was also recently reported in barramundi farmed in freshwater ponds in Thailand, suggesting this disease is not confined to marine aquaculture (Kerddee et al., 2020). Clinically affected fish show darkened bodies, scale loss, fin and tail erosion, and occasionally, cloudy eyes and red bellies (Gibson-Kueh et al., 2012; de Groof et al., 2015; Senapin et al., 2019; Nurliyana et al., 2020). Many of these gross abnormalities observed are similar to that caused by opportunistic bacterial infections, such that SDD was initially misdiagnosed as tenacibaculosis (Gibson-Kueh et al., 2012). Affected fish show systemic vasculitis and resulting tissue necrosis in all major organs, particularly in the spleen and kidney (Gibson-Kueh et al., 2012; Senapin et al., 2019).

Other emerging pathogens in cultured barramundi include *L. calcarifer* herpes virus (LCHV) (Chang et al., 2018; Meemetta et al., 2020), infectious spleen and kidney necrosis virus (ISKNV) (Dong et al., 2017a), *Vibrio harveyi* causing scale drop and muscle necrosis (Vh-SDMND) (Dong et al., 2017b), *L. calcarifer* birnavirus (LCBV) (Chen et al., 2019), and red sea bream iridovirus (RSIV) (Girisha et al., 2020). SDDV and LCHV infections in barramundi reportedly caused similar gross signs of scale loss (Chang et al., 2018), reiterating the need to use more specific diagnostic tests. PCR-based diagnostic methods have been established for these pathogens (Gias et al., 2011; de Groof et al., 2015; Meemetta et al., 2020; Sriisan et al., 2020). However, epidemiological studies characterizing the prevalence of these pathogens in farming sites have not yet been carried out. In fact, coinfections, rather than a single pathogen are commonly responsible for mortalities and production losses (Dong et al., 2015; Bastos Gomes et al., 2019; Nguyen et al., 2019; Kerddee et al., 2020). In some cases, such interactions might change how the host responds to a secondary infection in a counterintuitive manner, as for instance persistently nervous necrosis virus (NNV)-infected barramundi were shown to exhibit resistance to RSIV coinfection (Wu et al., 2013).

Recent genomic studies in barramundi have resulted in the development of linkage maps (Wang et al., 2015a, 2017a), molecular markers (Zhu et al., 2006a; Wang et al., 2015b), transcriptomes (Xia et al., 2011; Thevasagayam et al., 2015), and whole genome assemblies (Domingos et al., 2015; Vij et al., 2016). These studies have contributed to a better understanding of the biology of wild barramundi (Zhu et al., 2006b; Yue et al., 2009; Loughnan et al., 2019) and in aquaculture production (Domingos et al., 2013, 2014a,b, 2018, 2021; Loughnan et al., 2013, 2016; Ravi et al., 2014; Ngoh et al., 2015; Wang et al., 2015b). A number of studies have employed “omics” approaches targeted to better understand molecular pathways and genes involved in *L. calcarifer’s* adaptive stress response (Newton et al., 2013; Hook et al., 2017; Ma et al., 2020; Vij et al., 2020). Other studies investigated immune functions (Xia and Yue, 2010; Xia et al., 2011, 2013; Jiang et al., 2014; Liu et al., 2016), QTLs (Wang et al., 2017b) and disease resistance genes (Fu et al., 2013, 2014; Sun et al., 2020). Laboratory challenge trials have been carried out to understand the genetic basis of immune response and survival in barramundi associated with *V. harveyi* (Fu et al., 2013; Xia et al., 2011, 2013), *Photobacterium damselae* (Fu et al., 2013), *Streptococcus iniae* (Jiang et al., 2014), iridovirus (Wang et al., 2017b; Sun et al., 2020), and NNV (Liu et al., 2016). Transcriptome analyses based on RNAseq have proven to be a powerful tool to understand pathogenicity and fish immunity (Sudhagar et al., 2018).

To better understand recurring mortalities experienced during the early phases of sea cage culture of barramundi, this study investigated the transcriptional changes and associated histopathology in fish sampled during a major disease outbreak event. Tissues of clinically healthy and sick fish were collected, and histological analyses, qPCR, and PCR for significant pathogens and RNAseq methodologies were carried out. Our results showed significant changes in pathogen loads of SDDV but not LCHV, pathology, and differential gene expression between clinically healthy and sick barramundi.

## Materials and Methods

### Sample Collection

Juvenile barramundi were collected in a sea cage with a history of recent mortalities from a commercial farm in Singapore, in June 2019. Eight apparently diseased fish (hereafter termed “sick” fish—length, 26.5 ± 3.0 cm; weight, 231.3.1 ± 67.4 g) displaying lethargy (slow swimming at the surface), fin and body rot, and scale loss (or which scales were easily removed) were sampled for tissue collection, along with eight clinically healthy fish (hereafter termed “healthy”—length, 28.0 ± 2.5 cm; weight, 290.1 ± 71.6 g) exhibiting active swimming patterns, smooth body and skin, and clear eyes. Fish were euthanized in buckets containing 15 L of seawater and 15 ml of 10% clove oil (100 ppm), and immediately dissected after decapitation to collect the kidney, muscle, spleen, and liver (∼0.5 cm^3^). Tissues were subsampled and preserved either in RNAlater (Ambion, Austin, TX, United States) for RNA sequencing and pathogen screening by PCR and/or qPCR or 10% phosphate-buffered formalin for histology. Tissue samples in RNA later were kept on ice and transferred to a –20°C freezer on the same day for storage until processed for analyses. Formalin-fixed tissues were processed into 5 μm hematoxylin and eosin (H&E)-stained tissue sections at the Institute of Molecular and Cell Biology (IMCB) histology laboratory in Singapore. H&E-stained tissue sections were viewed under bright field microscopy, and images were captured using the Olympus Research Microscope BX53, Digital Camera DP74, and CellSens^TM^ Standard Imaging System (Olympus Corporation, Tokyo, Japan).”

### Screening of Five Putative Fish Pathogens by PCR Methods

The presence and load of pathogens in fish was identified *via* PCR/qPCR methods (Table 1). Firstly, genomic DNA was extracted from kidney and liver tissues of healthy (*n* = 8) and sick (*n* = 8) barramundi using a conventional phenol/chloroform and ethanol precipitation method. From this extract, 200 ng of DNA template was then used in each PCR reaction. Along with all tests for the target pathogen, amplification of the cytochrome *c* oxidase gene (COI) was included (Ivanova et al., 2007) to ensure quality of the DNA template. PCR diagnosis were conducted for three viral pathogens namely SDDV, LCHV, *Megalocytivirus*, and two bacterial pathogens *Tenacibaculum maritimum* and *Vibrio harveyi* causing scale drop and muscle necrosis (Vh-SDMND), as per Table 1.

**TABLE 1:** Summary of molecular tests employed to detect and/or quantify pathogens present in kidney and liver of barramundi.

**Pathogen**	**Method**	**Target**	**Reaction**	**Cycling conditions**	**Positive controls**	**References**
Scale drop disease virus (SDDV)	SYBR qPCR	SDDV *ATPase* gene	A 20-μl qPCR reaction contained the DNA template, 150 nM of each primer and 2 × KAPA SYBR FAST master mix (Kapa Biosystems, Inc., Wilmington, WA, United States)	95°C for 3 min and 40 cycles of 95°C for 3 s and 63°C for 30 s followed by melt curve analysis	DNA from SDDV-infected barramundi (Senapin et al., 2019)	Sriisan et al., 2020
*Lates calcarifer* herpes virus (LCHV)	SYBR qPCR	LCHV major envelop protein gene	A 20-μl qPCR reaction contained the DNA template, 200 nM of each primer and 1x iTaq Universal SYBR Green Supermix (Bio-Rad, Hercules, CA, United States)	95°C for 10 min and 40 cycles of 95°C for 10 s and 63°C for 30 s followed by melt curve analysis	DNA from LCHV-infected barramundi	Meemetta et al., 2020
*Megalocytivirus*	Single PCR	*Megalocytivirus* major capsid protein gene	A 25-μl PCR reaction contained the DNA template, 200 nM of each primer, 200 μM dNTP, 1.25 units of Taq DNA polymerase enzyme (RBC Bioscience, New Taipei City, Taiwan) and 1 × supplied buffer	94°C for 5 min and 35 cycles of 94°C for 30 s, 60°C for 30 s, and 72°C for 30 s, and a final extension step at 72°C for 5 min	DNA from *Megalocytivirus*-infected Asian sea bass (Dong et al., 2017a)	Gias et al., 2011
*Vibrio harveyi* causing scale drop and muscle necrosis (Vh-SDMND)	Duplex PCR	Vh-SDMND hypothetical protein gene and SDDV *ATPase* gene	A 25-μl PCR reaction contained the DNA template, 200 nM of each primer, and 1 × AccuStart II GelTrack PCR SuperMix (Quantabio, Beverly, MA, United States)	94°C for 3 min and 35 cycles of 94°C for 30 s, 60°C for 30 s and 72°C for 30 s, and an extension step at 72°C for 5 min	Plasmid harboring dual targets for both Vh-SDMND and SDDV	Taengphu et al., unpublished
*Tenacibaculum maritimum*	Single PCR	16S rDNA gene	A 20-μl PCR reaction contained the DNA template, 200 nM of each primer, 2 units of Taq polymerase (PCR Biosystems), and 1 × supplied buffer	94°C for 1 min and 35 cycles of 94°C for 30 s, 50°C for 30 s and 72°C for 1 min, and an extension step at 72°C for 5 min	Plasmid containing the *T. maritimum* insert target	Toyama et al., 1996

Copy numbers of SDDV and LCHV were calculated from respective standard graphs generated by qPCR amplifications of serially diluted plasmid containing corresponding viral insert target as previously described (Meemetta et al., 2020; Sriisan et al., 2020).

### Statistical Analyses

Assessment of statistical differences between sick and healthy fish viral loads in kidney and liver was performed with a Mann-Whitney *U*-test at a significance level of 0.05. The association between the viral loads and the observed histopathological scores (hepatic reserves, lipidosis, glomerulonephritis, and splenitis) was explored using PCA with Spearman rank metrics in Xlstat^®^ software (Addinsoft, Paris, France). The original set of eight variables of interest was reduced into two components of eigenvalues of 4.72 and 1.61 and represented as a two-dimensional plot.

### RNA Extraction, Library Preparation, and Sequencing

Total RNA was extracted from three immune competent tissues (liver, spleen, and kidney) and muscle from all 16 fish using an RNeasy^®^ Mini kit (Qiagen 74104, Qiagen, Frankfurt, Germany). All RNA samples were treated with RNase free DNase-I (M610A, Promega, Madison, WI, United States) to remove genomic DNA contamination. The quality and the quantity of the total RNA was determined with an Agilent 2100 Bioanalyzer (RNA 6000 Nano Chip Assay, Agilent, Böblingen, Germany) and a Qubit 3.0 (Quant-It dsRNA BR Assay, ThermoFischer Scientific, Waltham, MA, United States).

For the cDNA library preparations, 1 μg RNA was used as an input material for each sample. VAHTS mRNA-seq V3 Library Prep Kit for Illumina (NR611, Vazyme; San Diego, CA, United States) was used to generate sequencing libraries. In brief, mRNA with poly(A) was enriched by mRNA Capture Beads and fragmented by heating. Short mRNA was reverse-transcribed with random hexamer primers to generate the first cDNA, and then the second cDNA was synthesized. The cDNA fragments then went through an end repair process, the addition of a single “A” base to the 3′ end and then ligation of the adapters. The products were then purified and size selected (350 bp range). At the end, fragments were enriched by PCR and purified using VAHTSTM DNA Clean Beads. The quality and quantity of PCR product was determined by the Agilent Bioanalyzer 2100 and Qubit 2.0 (ThermoFisher). Finally, sequencing was undertaken on an Illumina Novaseq 6000 platform generating 150 bp paired-end reads.

### RNA-Seq Data Mapping, Gene Differential Expression, and Enrichment Analysis

To obtain high-quality clean data for downstream analyses, raw reads of FASTQ format were firstly processed through in-house perl scripts. In this step, the low-quality reads and reads containing adaptors or poly-*N* were removed. At the same time, Q20, Q30, GC content, and sequence duplication level of the clean data were calculated.

Reference genome and gene model annotation files of *L. calcarifer* were downloaded from the NCBI^1^ genome website directly. Index of the reference genome was built using Bowtie v2.2.3 (Langmead and Salzberg, 2012), and paired-end clean reads were aligned to the reference genome using TopHat v2.0.12 (Kim et al., 2013). The process of genetic quantification of gene expression level was carried out by HTSeq v0.6.1 by counting the read numbers mapped to each gene (Anders et al., 2015), and then the expected number of fragments per kilobase of transcript sequence per millions base pairs sequenced (FPKM) of each gene was calculated based on the length of the gene and reads count mapped to this gene.

Differential expression statistical analysis between organs from sick and healthy fish was performed using the DESeq R package (1.18.0) (Wang et al., 2010). The resulting *P*-values were adjusted using the Benjamini and Hochberg’s approach for controlling the false-discovery rate. Genes with an adjusted *P*-value < 0.05 found by DESeq were assigned as the threshold for indicating significantly differential expression. Gene Ontology (GO) enrichment analysis of differentially expressed genes was implemented by the GOseq R package, in which gene length bias was corrected (Young et al., 2010). GO terms with corrected *P*-values < 0.05 were considered significantly enriched by differentially expressed genes. KOBAS software (Mao et al., 2005) was utilized to test the statistical enrichment of those differential expression genes in KEGG^2^ pathways.

## Results

### Pathogen Screening Using PCR and qPCR

**FIGURE 1:**
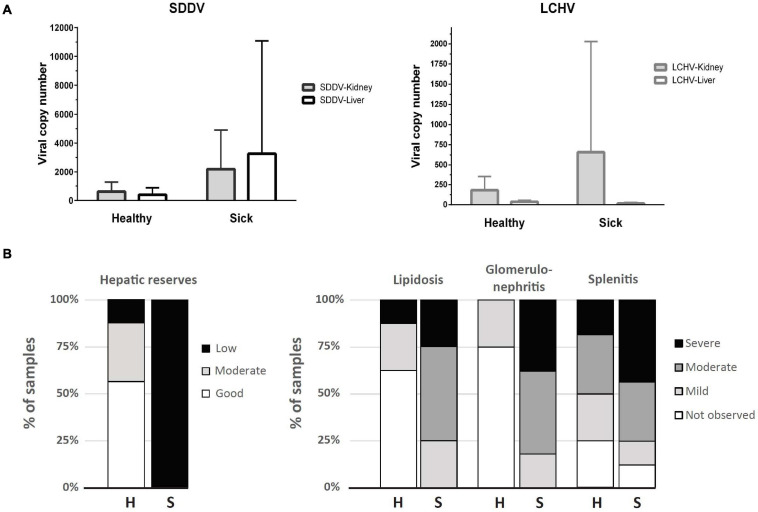
**(A)** Viral copy number (mean + SD) determined by qPCR assays for SDDV and LCHV of samples derived from kidney and liver (*n* = 8, except for liver of the sick fish, where *n* = 7). **(B)** Distribution of samples according to histopathology scores of liver (hepatic reserves and lipidosis), spleen (splenitis), and kidney (glomerulonephritis) of apparently healthy (H, *n* = 8) and sick (S, *n* = 8) barramundi.

While histopathology strongly suggested a primary infection with scale drop virus in both sick and clinically healthy fish, qualitative molecular tests (PCR) were carried out to rule out other possible concurrent infections that could cause the scale loss observed (Vh-SDMND, *T. maritimum*), or other viral diseases that could cause the occasional inclusion bodies observed in renal glomeruli or connective tissues within skeletal muscles in sick fish (*Megalocytivirus*). Based on PCR, liver and kidney samples returned negative for the presence of *Megalocytivirus*, Vh-SDMND, and *T. maritimum* in both sick and healthy fish, while all fish sampled were positive for SDDV and LCHV, with exception of two sick fish liver samples (out of seven) (Supplementary Table 1 and Supplementary Figure 1). SDDV and LCHV loads in the healthy fish ranged from 1 to 1,853 copies and 17 to 475 copies/qPCR reaction, respectively. SDDV and LCHV loads were higher in the sick fish, from 131 to 22,549 and 0 to 4,045 copies/qPCR reaction, respectively (Supplementary Table 2 and Figure 1A). SDDV overall loads were seven times higher than those for LCHV. SDDV loads in kidney and liver of sick fish were 8.4 and 3.6 times higher than those of healthy fish (Figure 1A). However, differences in loads between the two groups were not statistically significant (*P* > 0.05). The kidneys of sick fish had 3.6 times higher LCHV loads than those of healthy fish. Comparatively, LCHV loads in the liver were one magnitude lower than those in the kidney and two orders of magnitude lower than that of SDDV (Figure 1A, right). Amplicons from selected positive test samples (marked with an asterisk in Supplementary Table 1) were subjected to DNA sequence analysis and found to show one (99.3%) to zero nucleotide change (100% identity) among the SDDV product sequences. In contrast, two (97.85%) to no nucleotide differences (100% identity) were observed among the LCHV product sequences (Supplementary Figure 2).

### Histopathology

**FIGURE 2:**
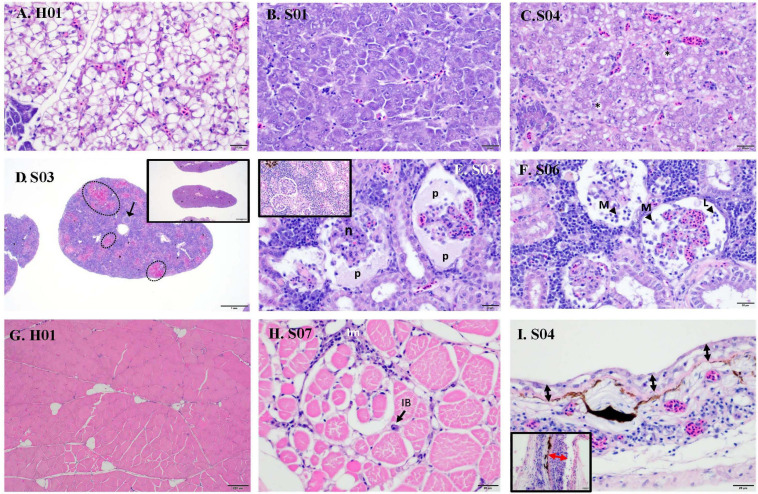
Histopathology of sick (S) and healthy (H) barramundi (hematoxylin and eosin stains). **(A)** Liver, wispy cytoplasmic appearance due to good levels of lipid reserves (fish H01). **(B)** Liver, homogenous, basophilic cytoplasm with low lipid reserves (fish S01). **(C)** Liver, severe hepatic lipidosis, with large lipid vacuoles or macrovesicles (*) (fish S04). **(D)** Spleen, moderate splenitis with multifocal necrosis (encircled, fish S03). Arrow shows occluded artery. Inset is spleen with no abnormalities (fish H06). **(E)** Kidney, severe glomerulonephritis, with pyknosis and karyorrhexis [necrosis (n)] and protein effusion (p) into Bowman’s space (fish S03). Inset is kidney with no abnormalities (fish H01). **(F)** Kidney, severe glomerulonephritis with glomerular necrosis and infiltration into Bowman’s space of macrophages (M) and lymphocytes (L) (fish S06). **(G)** Normal skeletal muscles (fish H01). **(H)** Interstitial myositis (Im), associated with occasional viral inclusion body (IB) (fish S07). **(I)** Thinning of epidermis (black arrow) (Fish S04), compared with thicker epidermis (red arrow) (inset, fish H01). Lymphocytic-monocytic infiltration surrounding dermal blood vessels.

Raw data on individual viral loads and histopathology scores are presented in Supplementary Table 2. Overall, clinically sick fish showed a greater degree of abnormalities, often related to inflammatory processes (Figure 1B). The wispy cytoplasmic appearance of H&E-stained livers with good hepatocellular reserves in healthy fish, differs from the more homogenous, basophilic cytoplasm in sick fish, with depleted hepatic reserves (Figures 2A,B). Moderate (score 2) to good (score 3) hepatocellular lipid and glycogen reserves were observed in most of the livers of clinically healthy barramundi (Figures 1B, 2A), in contrast to low hepatic reserves observed in all sick fish (Figures 1B, 2B). Moderate to severe, accumulation of large, round, lipid vacuoles or macrovesicles in liver cells (lipidosis), was observed in most sick barramundi (Figures 1B, 2C). Lipidosis is either not observed or mild in healthy fish, with the exception of one individual which had the highest SDDV loads within this group (Figure 1A and Supplementary Table 2).

Moderate to severe splenic inflammation (splenitis) were observed in both healthy and sick fish (Figure 1B). Moderate splenitis is characterized by multifocal necrosis (Figure 2D), while severe splenitis has multifocal to coalescing necrosis, affecting extensive areas of the spleen. Spleens with multifocal infarcts, or tissue deaths (necrosis), had occluded blood vessels, due to marked inflammatory response in the endothelium (obliterative endarteritis) (Figure 2D). While there was splenitis in a significant number of both clinically healthy and sick fish, moderate to severe kidney inflammation (glomerulonephritis) was observed only in all sick fish. The kidneys of diseased barramundi showed glomerular necrosis, protein effusion, and mixed infiltration of macrophages and lymphocytes into the Bowman’s space (protein loosing, necrotizing glomerulonephritis) (Figures 2E,F).

Mild multifocal interstitial myositis was observed in both healthy (H02, H04, and H05) and sick fish (S07), and occasionally in association with the presence of inclusion bodies (Figure 2H). Moderate, diffuse dermatitis was observed where skin was intact in tissue sections examined, in both healthy (H01, H02, H04, and H06) and sick fish (S02 and S04). In addition, severe thinning of epidermis was observed in the sick fish S04, lymphocytic-monocytic infiltration surrounding dermal blood vessels (perivasculitis) and occluded dermal blood vessels with obliterative endarteritis was observed in sick fish S06 (Figure 2I).

**FIGURE 3:**
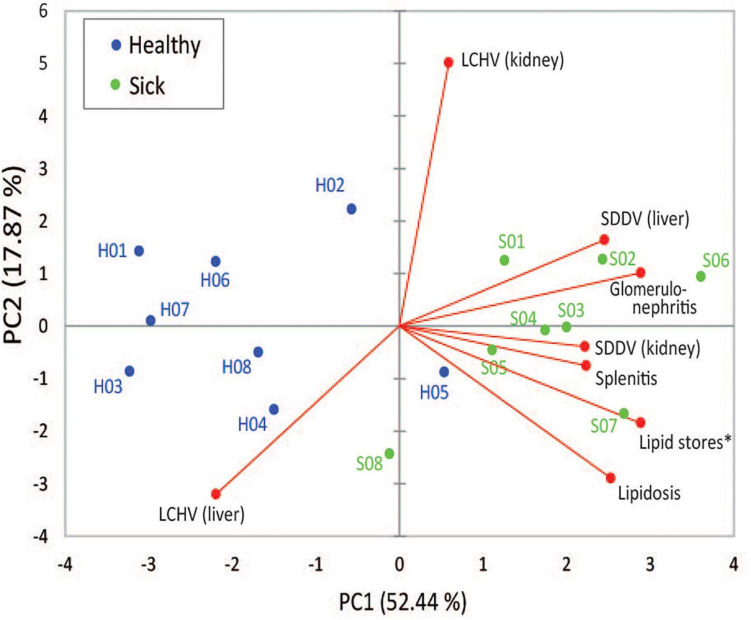
First and second components of principal component analysis (PCA), showing the relationship among SDDV and LCHV loads and histopathology features (red dots) and the overlay distribution of the individual sample of healthy (H01–H08, blue dots) and sick (S01–S08, green dots) barramundi juveniles in this study. Asterisk, for graphical purposes, lipid store values were multiplied by –1.

Two principal components derived from PCA explained 70.3% of the variance among SDDV and LCHV viral loads and histopathological findings (Figure 3). A positive association was observed between SDDV loads (both in the liver and in the kidney) and the major negative histopathological findings (glomerulonephritis, splenitis, and lipidosis) (component F1 > 2), which were inversely related to lipid score (component F1 < –2). In contrast, the PCA revealed no direct association of LCHV loads with any of the histopathological findings (and neither with SDDV loads). Although all healthy individuals were positive for both viruses (albeit with lower loads than sick individuals) and did present some of the histopathological alterations (most notably on the spleen, Figure 1B), the PCA revealed a marked difference in grouping of sick (F1 > 0) and healthy (F1 < 0) fish, with exception of S08 and H05 individuals, which respectively had the lowest and the highest SDDV loads within their groups (Supplementary Table 2).

### Severe RNA Degradation Observed in Spleen and Kidney of Sick Fish

**FIGURE 4:**
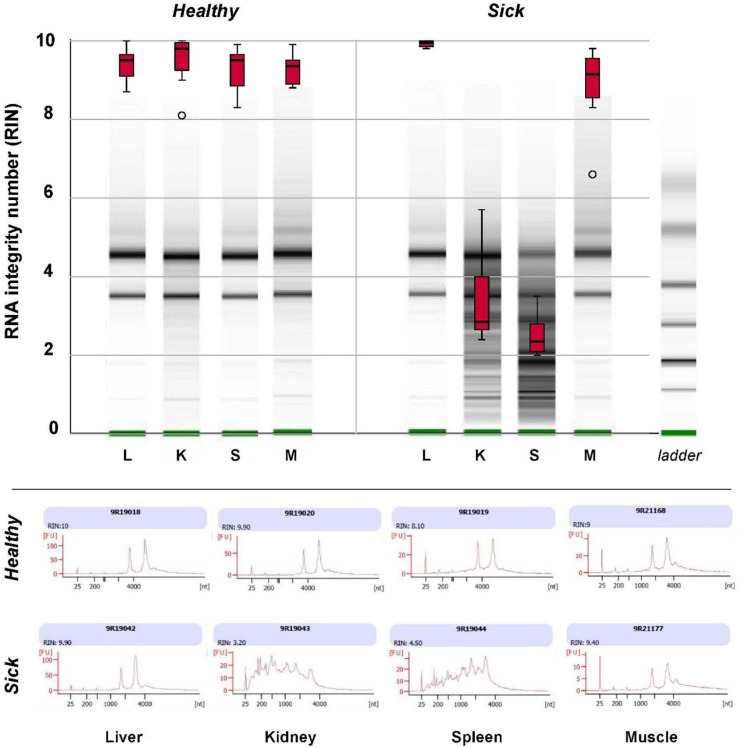
Box plots of RNA integrity number (RIN) of total RNA extracted from liver (L), kidney (K), spleen (S), and muscle (M) tissues of eight “sick” and eight “healthy” juvenile barramundi from a sea cage farm, as determined with an Agilent 2100 Bioanalyzer RNA 6000. Nanochip assay with representative gel images (top) and electropherogram (bottom) of total RNA extracts of sick and healthy fish, indicated severe degradation in RNA extracted from kidneys and spleens of sick fish.

Major differences were observed in the quality of RNA extracted from spleen and kidney of healthy vs. sick fish and between different organs in sick fish, as assessed with an Agilent 2100 Bioanalyzer (Figure 4). RNA integrity number (RIN, mean ± S.D.) for extracts from spleen and kidney of sick fish were 2.50 ± 0.55 and 3.39 ± 1.15, respectively. This contrasts with RIN of 9.93 ± 0.09 for liver, and 8.86 ± 1.03 for muscle RNA extracts for sick fish, and 9.37 ± 0.11 for RNA derived from all organs of healthy fish. As RNA in spleen and kidney samples of sick fish were degraded and did not pass quality control for sequencing, only liver and muscle tissues were used for transcriptomic comparisons between the two groups.

### Liver and Muscle RNA Sequencing, Mapping, and Analysis of Differentially Expressed Genes

RNA sequencing performed on eight sick and eight healthy fish generated a total of 1,426.10 and 1,831.68 million clean reads, from the liver and muscle tissues, respectively. An average of 101.80 million clean reads (ranging from 82.48 to 167.31 million) was obtained from each sample. The Q30 (i.e., probability of an incorrect base call of 1 in 1,000) and GC percentages of the reads were 92.84 and 50.08%, respectively. All the filtered clean reads were mapped individually against the annotated genome of *L. calcarifer*. In total, 2,504.23 million reads were successfully mapped, with approximately 2,306.56 million reads (92.1%) uniquely mapped (i.e., mapped to a single locus in the genome). Detailed sequencing and mapping results are summarized in Supplementary Table 3. RNAseq data (32 trancriptomes) have been deposited in NCBI under the accession number PRJNA713978.

**FIGURE 5:**
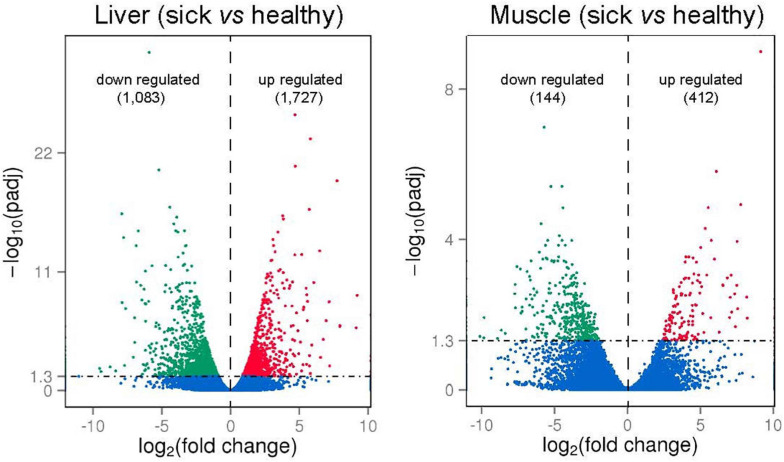
Volcano plots of differently expressed genes (DEGs; numbers up- or downregulated in brackets) in liver and muscle of sick vs. healthy barramundi. *x*-axis represents log_2_-transformed fold change and the *y*-axis indicates –log_10_-transformed adjusted significance. Each dot indicates an individual gene that was significantly upregulated (red), downregulated (green), and non-differentially regulated (blue). The horizontal line represents adjusted *P*-value (*P* < 0.05 cutoff).

To identify gene expression changes between liver (or between muscle) of sick and healthy fish, the FPKM method was used to calculate the expression levels of genes (Figure 5). For liver tissues, the average Pearson correlation coefficient (*R*^2^ values) for gene expression values was 0.92 (0.89–0.97) in healthy fish and 0.95 (0.93–0.98) in sick fish, indicating the high repeatability of biological replicates. For muscle tissues, the average *R*^2^ values was 0.82 (0.71–0.94) in healthy fish and 0.80 (0.51–0.95) in sick fish, which suggested less similarity in the gene expression patterns of muscle relative to liver between sick and healthy fish. Given the high number of biological samples randomly sampled within each group (*n* = 8) for a transcriptomic study, but still limited in relation to the farmed population subjected to the disease outbreak, all samples were utilized in the analysis. Comparison between liver of two groups (sick vs. healthy fish) revealed 2,810 significantly differentially expressed genes (DEGs). Among them, 1,083 were upregulated and 1,727 were downregulated in the liver of sick fish (Figure 5 and Supplementary Table 4). In the muscle, a total of 556 significantly DEGs were discovered, with 144 genes upregulated and the rest downregulated in sick fish (Figure 5 and Supplementary Table 5). To illustrate the DEGs detected in sick and healthy fish, heatmaps were generated for both liver and muscle separately (Supplementary Figures 3A,B). The transcriptomic profile in the livers of healthy fish was obviously different from that of the sick fish, with all eight samples from each group contained within two main clusters (Supplementary Figure 3A). Whereas in muscle, the expression pattern of the DEGs showed a less distinct clustering between the two groups compared with that of liver, with some sample overlap between muscle of sick and healthy animals between the two main clusters (Supplementary Figure 3B), probably because muscle is not an immune-related organ like liver and is thus is less affected by the disease. About 32% of the DEGs found in the muscle (*n* = 176) were also differentially expressed in the liver, whereby a 97% in concordance between up- or downregulation was observed. In terms of gene ontology, DEGs in both tissues were primarily classified within “cellular component,” followed by “biological processes” and then “molecular function” (Supplementary Figure 4).

The top 20 significantly up− or downregulated DEGs identified in liver and muscle are listed in Tables 2, 3, respectively. A number of these genes were related to inflammatory and immune responses of juvenile barramundi to viral infections. Upregulated gene expression in liver of sick fish included interleukin-1 receptor type 2-like (il-1r2l), cytokine-like protein 1 (cytl1), toll-like receptor 13 (tlr13), hepatitis A virus cellular receptor 1 homolog (havcr-1), and leucine-rich repeat-containing protein 52-like (lrrc52), whereas the complement C1q-like protein 4 (c1ql4), complement C1q tumor necrosis factor-related protein 1-like (ctrp1), phospholipase A2-like (pla2s), and retinol-binding protein 2 (rbp2) were downregulated (Table 2). In muscle of sick fish, vital components of innate immunity, including toll-like receptor 13 (tlr13) and toll-like receptor 5 (tlr5), Fc receptor-like protein 5 (fcrl5), and interleukin-1 receptor type 2-like (il-1r2) were significantly upregulated. In contrast, interferon-induced protein 44-like (ifi44l), E3 ubiquitin-protein ligase TRIM21-like (trim21), complement C1q tumor necrosis factor-related protein 1-like (ctrp1), golgi-associated plant pathogenesis-related protein 1-like (gapr-1), and G protein-coupled receptor 17 (gpr17), associated to immune response were downregulated in the muscle of sick fish (Table 3).

**TABLE 2:** The top 20 significantly up- or downregulated genes in liver of sick barramundi.

**Gene ID/name**	**Log_2_ fold change**	***P* (adj)**	**Up/downregulation**	**Gene description**
LOC108880246	9.21	1.44E-09	Up	Excitatory amino acid transporter 5-like
LOC108884404	7.94	1.26E-06	Up	Fibroblast growth factor 21
slc7a11	7.94	1.03E-06	Up	Solute carrier family 7 member 11
LOC108897018	7.74	3.82E-20	Up	Interleukin-1 receptor type 2-like
LOC108893249	7.20	0.00550	Up	Cytokine-like protein 1
LOC108874878	7.17	6.74E-09	Up	Solute carrier family 1 member 8b
LOC108885470	6.95	3.63E-07	Up	Hepatitis A virus cellular receptor 1 homolog
LOC108879612	6.74	1.95E-05	Up	Insulin-like growth factor binding protein 1a
LOC108879014	6.55	0.00090	Up	Leucine-rich repeat-containing protein 52-like
LOC108897016	5.91	6.64E-05	Up	Interleukin-1 receptor type 2-like
LOC108887824	5.80	0.02790	Up	Hepcidin-like
LOC108885100	5.80	5.26E-24	Up	Toll-like receptor 13
LOC108902039	5.74	0.00060	Up	Polycystic kidney disease 1b
LOC108890793	5.74	0.00550	Up	IgGFc-binding protein-like
LOC108874726	5.72	1.68E-17	Up	Neoverrucotoxin subunit alpha-like
LOC108889506	5.60	6.07E-09	Up	Cytosolic sulfotransferase 3-like
nim1k	5.57	3.70E-07	Up	NIM1 serine/threonine protein kinase
LOC108885837	5.54	1.27E-09	Up	Protein NDRG1-like
Tub	5.53	0.00389	Up	TUB bipartite transcription factor
LOC108902348	5.50	3.15E-10	Up	Ladderlectin-like
LOC108891007	–6.21	9.61E-09	Down	Complement C1q-like protein 4
LOC108890805	–6.25	0.01456	Down	Protein jagged-1a-like
LOC108881856	–6.28	3.94E-10	Down	P17/29C-like protein DDB_G0287399
abcg4	–6.34	0.00404	Down	ATP-binding cassette, subfamily G (WHITE), member 4a
paqr5	–6.41	0.03062	Down	Progestin and adipoQ receptor family member Vb
LOC108891005	–6.45	4.60E-09	Down	Complement C1q-like protein 4
LOC108878005	–6.49	0.04627	Down	Neuropeptide Y receptor Y8b
LOC108887476	–6.54	1.73E-10	Down	Cytochrome P450 2K1-like
LOC108879156	–6.70	1.65E-15	Down	Granzyme E-like
LOC108893889	–6.84	8.61E-11	Down	High choriolytic enzyme 1-like
LOC108890833	–6.85	3.83E-14	Down	Solute carrier family 12 member 3-like
LOC108882788	–6.97	3.75E-05	Down	Glutamate receptor, ionotropic, AMPA 1a
LOC108899398	–6.98	1.96E-07	Down	Fatty acid binding protein 10a, liver basic
LOC108881836	–7.07	0.00086	Down	Semaphorin 5A
LOC108878838	–7.63	2.81E-08	Down	Chymotrypsin-like elastase family member 3B
LOC108880291	–7.88	7.82E-09	Down	Phospholipase A2-like
LOC108890739	–7.91	4.23E-17	Down	Complement C1q tumor necrosis factor-related protein 1-like
LOC108876188	–8.35	0.01616	Down	Mid1-interacting protein 1-B-like
LOC108899606	–9.42	0.01790	Down	L-Rhamnose-binding lectin SML-like
rbp2	–9.53	0.00918	Down	Retinol-binding protein 2

**Given in descending order of log_2_ fold change*.*

**TABLE 3:** The top 20 significantly up- or downregulated genes in muscle of sick barramundi.

**Gene ID/name**	**Log_2_ fold change**	***P* (adj)**	**Up/downregulation**	**Gene description**
LOC108885100	9.14	9.88E-10	Up	Toll-like receptor 13
acod1	8.21	0.01243	Up	Aconitate decarboxylase 1
LOC108874726	7.77	1.16E-05	Up	Neoverrucotoxin subunit alpha-like
donson	7.55	0.00698	Up	DNA replication fork stabilization factor DONSON
LOC108873250	7.52	0.00011	Up	Granulocyte colony-stimulating factor-like
LOC108888912	7.50	0.00171	Up	Toll-like receptor 5
chrng	7.40	0.02103	Up	Cholinergic receptor, nicotinic, gamma
LOC108880557	7.35	0.00275	Up	Fc receptor-like protein 5
LOC108893722	7.15	0.00854	Up	Mucin-5AC-like
LOC108897018	7.04	0.00344	Up	Interleukin-1 receptor type 2-like
LOC108884001	7.01	0.01199	Up	Protein OSCP1-like
LOC108890809	6.55	0.00172	Up	Hydroxycarboxylic acid receptor 2-like
LOC108880702	6.54	0.00167	Up	Carcinoembryonic antigen-related cell adhesion molecule 20-like
LOC108894065	6.29	0.02952	Up	Matrix metallopeptidase 13a
gadl1	6.09	1.56E-06	Up	Glutamate decarboxylase like 1
LOC108884332	5.74	0.00011	Up	Acyl-coenzyme A thioesterase 2, mitochondrial-like
lrriq1	5.72	0.01210	Up	Leucine-rich repeats and IQ motif containing 1
LOC108886122	5.54	1.40E-05	Up	Arginase 1
LOC108883738	5.433	0.00090	Up	Carboxypeptidase N subunit 2
alpl	5.335	5.08E-05	Up	Alkaline phosphatase, biomineralization associated
lhx8	–6.40	0.04081	Down	LIM homeobox 8
foxe1	–6.42	0.00211	Down	Forkhead box E1
cd248	–6.50	0.00630	Down	CD248 molecule
dlx6	–6.52	0.03813	Down	Distal-less homeobox 6a
LOC108900437	–6.55	0.00378	Down	Tissue alpha-L-fucosidase-like
LOC108890438	–6.61	0.00038	Down	Nuclear factor 7, ovary-like
folh1b	–6.67	0.00124	Down	Folate hydrolase 1B
LOC108877493	–6.87	0.00073	Down	EF-hand and coiled-coil domain-containing protein 1-like
LOC108892256	–6.95	0.00378	Down	dickkopf-related protein 2-like
LOC108890833	–6.97	0.02433	Down	Solute carrier family 12 member 3-like
LOC108893465	–7.23	0.00627	Down	von Willebrand factor A domain-containing protein 7-like
LOC108874532	–7.23	0.00811	Down	Interferon-induced protein 44-like
gpr17	–7.35	0.00062	Down	G protein-coupled receptor 17
LOC108889688	–7.68	0.00657	Down	Growth arrest-specific 2b
LOC108890725	–7.71	0.00162	Down	E3 ubiquitin-protein ligase TRIM21-like
mei1	–8.39	0.03185	Down	Meiotic double-stranded break formation protein 1
LOC108896482	–8.64	0.02613	Down	Secretory calcium-binding phosphoprotein 7
LOC108878074	–8.92	0.04369	Down	Golgi-associated plant pathogenesis-related protein 1-like
LOC108897956	–9.83	0.01199	Down	Calpain-2 catalytic subunit-like
LOC108890739	–10.12	0.038363	Down	Complement C1q tumor necrosis factor-related protein 1-like

**Given in descending order of log_2_ fold-change*.*

### Immune-Relevant Pathways Identified in Liver and Muscle of Farmed Barramundi

Based on the DEG findings in sick vs. healthy fish, we performed KEGG pathway classification and functional enrichment analysis. Liver DEGs were classified into a total of 141 pathways, whereby 42 pathways showed statistical significance (corrected *P*-value < 0.05) (Supplementary Table 6). Muscle DEGs were classified into 69 pathways, whereby four pathways were statistically significant (corrected *P*-value < 0.05) (Supplementary Table 7). The top 20 enriched KEGG pathways in the liver and muscle in relation to their rich factor (i.e., the ratio of DEG numbers to all gene numbers annotated in a pathway) are shown in Supplementary Figures 5, 6, respectively.

Furthermore, a total of 27 pathways relevant to innate and adaptive immunity function were identified based on 525 DEGs in the liver, whereby 10 pathways were significantly enriched. Out of those 27 immune-related pathways, 22 were also shared by 127 DEGs in the muscle, whereby four of those pathways were significantly enriched (Table 4). Based on the significance value, the phagosome was the most enriched pathway in the liver, whereas the shared ECM-receptor interaction was the most enriched pathway in the muscle. Most of these immune relevant pathways (16) were classified under environmental information processing, including cytokine-cytokine receptor interaction, calcium signaling pathway, ECM-receptor interaction, cell adhesion molecules (CAMs), neuroactive ligand-receptor interaction, and MAPK signaling pathways. According to the KEGG organismal immune system, five pathways were identified: intestinal immune network for IgA production, Toll-like receptor, cytosolic DNA-sensing, NOD-like receptor, and RIG-I-like receptor signaling pathways. In addition, DEGs were also classified under the broader pathways of endocrine and metabolic disease, infectious diseases (viral and bacterial), cell growth and death, and xenobiotics biodegradation and metabolism (Table 4).

**TABLE 4:** Distribution of the differentially expressed genes in immune-relevant pathways in sick (vs. healthy) barramundi.

**KEGG pathway**	**Liver**		**Muscle**		**Pathway ID**	**Pathway subclass**
	**DEG**	**Corr. *P*-value**	**DEG**	**Corr. *P*-value**		
Phagosome	41	5.39E-08	4	*	Ko04145	Transport and catabolism
Cytokine-cytokine receptor interaction	35	3.06E-05	6	*	Ko04060	Signaling molecules and interaction
Calcium signaling pathway	46	3.92E-05	10	*	Ko04020	Signal transduction
ECM-receptor interaction	21	0.000141	13	1.41E-07	Ko04512	Signaling molecules and interaction
Cell adhesion molecules (CAMs)	29	0.000141	7	*	Ko04514	Signaling molecules and interaction
Focal adhesion	38	0.001475	17	2.81E-05	Ko04510	Cellular community
Neuroactive ligand-receptor interaction	53	0.003021	20	0.000240	Ko04080	Signaling molecules and interaction
AGE-RAGE signaling pathway in diabetic complications	22	0.005143	4	*	Ko04933	Endocrine and metabolic disease
Intestinal immune network for IgA production	11	0.005172	2	*	Ko04672	Immune system
Insulin signaling pathway	25	0.019897	1	*	Ko04910	Endocrine system
MAPK signaling pathway	35	*	13	0.033187	Ko04010	Signal transduction
Wnt signaling pathway	18	*	7	*	Ko04310	Signal transduction
Herpes simplex infection	21	*	1	*	Ko05168	Infectious diseases: Viral
mTOR signaling pathway	21	*	4	*	Ko04150	Signal transduction
Apoptosis	15	*	3	*	Ko04210	Cell growth and death
Lysosome	14	*	3	*	Ko04142	Transport and catabolism
PPAR signaling pathway	13	*	1	*	Ko03320	Endocrine system
TGF-beta signaling pathway	11	*	2	*	Ko04210	Signal transduction
Ubiquitin mediated proteolysis	10	*	1	*	Ko04120	Folding, sorting and degradation
Toll-like receptor signaling pathway	10	*	1	*	Ko04620	Immune system
Salmonella infection	10	*	1	*	Ko05132	Infectious disease: bacterial
p53 signaling pathway	6	*	3	*	Ko04115	Cell growth and death
Drug metabolism—other enzymes	6	*	0	*	Ko00983	Xenobiotics biodegradation and metabolism
Drug metabolism—cytochrome P450	6	*	0	*	Ko00982	Xenobiotics biodegradation and metabolism
Cytosolic DNA-sensing pathway	2	*	0	*	Ko04623	Immune system
NOD-like receptor signaling pathway	4	*	0	*	Ko04621	Immune system
RIG-I-like receptor signaling pathway	2	*	0	*	Ko04622	Immune system

* Corr. P-value > 0.05.

### Key DEGs Related to Immune Responses Are Mostly Downregulated in Liver and Muscle of Sick Barramundi

DEGs associated to immune-relevant pathways are listed in Supplementary Table 8 (liver) and Supplementary Table 7 (muscle), whereas DEGs, further classified by immune relevant gene families, are presented in Table 5. About 65 and 89% of these DEGs were downregulated in the liver and in the muscle, respectively. Most genes in Table 5 are related to innate immunity: pattern recognition receptors (PRRs) including Toll-like receptors (tlr5, tlr13) and C-type lectin receptors (cl-11l, colec12, and selp); inflammatory cytokines and receptors including interleukins (il-1β, irak4, and il11a) and interleukin receptors (il13rα1, il17ra, and il1r1l), TNF including (tnfrsf11a, tnfaip3, tnfsf10l, and tnfsf12); chemokines (ccl4, ccl25b, and c-x-cl12a) and chemokines receptors (ccr7, cxcr3l, ccr6b, and ackr3b); complement factors (C3l and C1q); collagen (col4a5, col6a6, col6a3, col1a1a, col1a1b, and col2a1b); and myd88 as innate immune signal transduction adaptor CD molecules. In addition, gene families related to the adaptive immune response, identified only in the liver, such as B cell receptor CD22-like (down), and T cell tcirg1 (up) and nfatc1 (down), and major histocompatibility complex (MHC) such as h2-aa, h2-eb1, and hla-dap1 were significantly downregulated (except for tcirg1) in sick barramundi.

**TABLE 5:** Summary of immune-relevant genes characterized from sick (vs. healthy) barramundi based on KEGG functional analysis.

**Gene family**	**Gene name**	**Liver**			**Muscle**			**Gene description**	**Gene ID**
		**Log_2_ fold change**	***P* (adj)**	**Up/downregulated**	**Log_2_ fold change**	***P* (adj)**	**Up/downregulated**		
Toll-like receptor	tlr5	2.83	0.012439	Up	7.50	0.00171	Up	Toll-like receptor 5	LOC108888912
	tlr13	5.80	5.26E-24	Up	9.14	9.88E-10	Up	Toll-like receptor 13	LOC108885100
Lectin	cl-11l	–1.33	0.004857	Down	–^a^	–^a^	–^a^	Collectin-11-like	LOC108889727
	colec12	2.60	0.00014	Up	–^a^	–^a^	–^a^	Collectin subfamily member 12	LOC108883269
	selp	2.76	2.82E-06	Up	–^a^	–^a^	–^a^	Selectin P	LOC108877420
Complement	C1ql4	–6.21	9.61E-09	Down	–^a^	–^a^	–^a^	Complement C1q-like protein 4	LOC108891007
	C3	–2.51	0.001909	Down	–^a^	–^a^	–^a^	Complement C3-like	LOC108885851
	C5	1.59	0.001243	Up	–^a^	–^a^	–^a^	Complement C5	C5
	C6	3.24	1.41E-13	Up	–^a^	–^a^	–^a^	Complement C6	C6
CTRP	ctrp1	–7.91	4.23E-17	Down	–10.12	0.038363	Down	Complement C1q tumor necrosis factor-related protein 1-like	LOC108890739
Chemokine	ccl4	–3.15	0.000577	Down	–^a^	–^a^	–^a^	C-C motif chemokine 4 homolog	LOC108890197
	ccl25b	0.92	0.04234	Up	–^a^	–^a^	–^a^	Chemokine (C–C motif) ligand 25b	LOC108881384
	c-x-cl12a	0.99	0.031141	Up	–^a^	–^a^	–^a^	Chemokine (C–X–C motif) ligand 12a (stromal cell-derived factor 1)	LOC108888279
	ccr7	–^a^	–^a^	–^a^	–2.93	0.046610	Down	Chemokine (C–C motif) receptor 7	ccr7
	cxcr3l	1.23	0.007315	Up	–^a^	–^a^	–^a^	C–X–C chemokine receptor type 3-like	LOC108880701
	ackr3b	1.09	0.015943	Up	–^a^	–^a^	–^a^	Atypical chemokine receptor 3b	ackr3
	ccr6b	–1.77	0.000066	Down	–^a^	–^a^	–^a^	Chemokine (C–C motif) receptor 6b	LOC108896100
Interleukin	il-1β	3.91	5.09E-06	Up	–^a^	–^a^	–^a^	Interleukin-1 beta-like	LOC108878374
	irak4	1.24	0.007064	Up	–^a^	–^a^	–^a^	Interleukin-1 receptor-associated kinase 4	irak4
	il11a	3.08	0.000064	Up	–^a^	–^a^	–^a^	Interleukin 11a	LOC108887322
	il13rα1	1.75	0.000044	Up	–^a^	–^a^	–^a^	Interleukin 13 receptor, alpha 1	LOC108882024
	il1r1l	1.02	0.031425	Up	–^a^	–^a^	–^a^	Interleukin-1 receptor type 1-like	LOC108897039
	il17ra	–1.33	0.031206	Down	–^a^	–^a^	–^a^	Interleukin-17 receptor A-like	LOC108888530
	Il7R-αl	–1.37	0.002399	Down	–^a^	–^a^	–^a^	Interleukin-7 receptor subunit alpha-like	LOC108892301
Tumor necrosis factor (TNF)	tnfrsf11a	1.89	0.013310	Up	–^a^	–^a^	–^a^	Tumor necrosis factor receptor superfamily, member 11a, NFKB activator	LOC108900798
	tnfaip3	1.78	0.000031	Up	–^a^	–^a^	–^a^	Tumor necrosis factor, alpha-induced protein 3	tnfaip3
	tnfsf10l	–2.05	0.003045	Down	–^a^	–^a^	–^a^	Tumor necrosis factor ligand superfamily member 10-like	LOC108885067
	tnfsf12	–2.54	0.000000	Down	–3.86	0.002753	Down	TNF superfamily member 12	LOC108902465
Collagen	col4a5	–3.78	1.87E-08	Down	–^a^	–^a^	Down	Collagen alpha-5 (IV) chain-like	LOC108883501
	col6a6	–1.78	0.046855	Down	–5.04	0.000399	Down	Collagen alpha-6 (VI) chain-like	LOC108881847
	col6a6	–3.93	0.000086	Down	–4.43	0.001080	Down	Collagen alpha-6 (VI) chain-like	LOC108881842
	col6a3	–1.52	0.027935	Down	–^a^	–^a^	Down	Collagen type VI alpha 3 chain	col6a3
	col1a1b	–3.32	0.000073	Down	–3.63	0.004069	Down	Collagen, type I, alpha 1b	LOC108876589
	col1a2	–3.80	0.000046	Down	–3.69	0.003305	Down	Collagen, type I, alpha 2	col1a2
	col6a1	–1.75	0.000495	Down	–3.79	0.001714	Down	Collagen, type VI, alpha 1	col6a1
	col6a2	–2.18	1.56E-06	Down	–2.81	0.046610	Down	Collagen, type VI, alpha 2	LOC108883902
	col2a1b	–^a^	–^a^	Down	–2.96	0.036736	Down	Collagen type II alpha 1b	LOC108884852
	col1a1a	–3.94	0.000021	Down	–4.67	0.007110	Down	Collagen, type I, alpha 1a	LOC108898561
	col6a6	–^a^	–^a^	Down	–3.82	0.005404	Down	Collagen, type VI, alpha 6	col6a6
Glutathione *S*-transferase (gst)	gstm3l	–1.22	0.004676	Down	–^a^	–^a^	–^a^	Glutathione *S*-transferase Mu 3-like	LOC108876447
	gsta.1	–1.96	0.038464	Down	–^a^	–^a^	–^a^	Glutathione *S*-transferase, alpha tandem duplicate 1	LOC108891126
	mgst3a	–1.28	0.002452	Down	–^a^	–^a^	–^a^	Microsomal glutathione S-transferase 3a	mgst3a
CD molecules	myd88	1.15	0.010149	Up	–^a^	–^a^	–^a^	MYD88 innate immune signal transduction adaptor	myd88
	cd276	1.27	0.008465	Up	–^a^	–^a^	–^a^	CD276 molecule	cd276
	cd74	–1.64	0.000159	Down	–^a^	–^a^	–^a^	CD74 molecule	cd74
	cd276	–1.05	0.038810	Down	–^a^	–^a^	–^a^	CD276 antigen-like	LOC108886627
	cd34	–^a^	–^a^	–^a^	–3.89	0.018911	Down	CD34 molecule	cd34
	cd166	–^a^	–^a^	–^a^	–2.95	0.021215	Down	CD166 antigen homolog A-like	LOC108899782
	cd248	–^a^	–^a^	–^a^	–6.50	0.00630	Down	CD248 molecule	cd248
B cell	cd22	–1.15	0.017344	Down	–^a^	–^a^	–^a^	B-Cell receptor CD22-like	LOC108880790
T cell	tcirg1	1.25	0.006142	Up	–^a^	–^a^	–^a^	T-Cell immune regulator 1	tcirg1
	nfatc1	–1.97	0.010264	Down	–^a^	–^a^	–^a^	Nuclear factor of activated T cells 1	LOC108899182
Major histocompatibility complex (MHC)	h2-aa	–2.37	0.027046	Down	–^a^	–^a^	–^a^	H-2 class II histocompatibility antigen, A-U alpha chain-like	LOC108892329
	h2-eb1	–1.60	0.016974	Down	–^a^	–^a^	–^a^	H-2 class II histocompatibility antigen, E-D beta chain-like	LOC108890468
	h2-eb1	–1.97	0.008825	Down	–^a^	–^a^	–^a^	H-2 class II histocompatibility antigen, E-S beta chain-like	LOC108892328
	h2-eb1	–2.62	6.93E-06	Down	–^a^	–^a^	–^a^	H-2 class II histocompatibility antigen, E-S beta chain-like	LOC108882204
	h2-eb1	–3.02	0.000078	Down	–^a^	–^a^	–^a^	H-2 class II histocompatibility antigen, E-S beta chain-like	LOC108882336
	hla-dap1	–1.67	0.000442	Down	–^a^	–^a^	–^a^	HLA class II histocompatibility antigen, DP alpha 1 chain-like	LOC108882210

**^*a*^No differential expression of the gene in liver or muscle*.*

## Discussion

Barramundi farming in Southeast Asia has been severely affected by disease outbreaks. In Singapore, where this particular study has taken place, farmers have reported mass mortalities of juveniles during the transition between nursery phases (∼50 to 500 g) and the final grow out period as a recurring phenomenon over the last few years. Although several novel viral pathogens (SDDV, LCHV, LCBV) affecting barramundi have been first reported and identified in Singaporean farms (Gibson-Kueh et al., 2012; de Groof et al., 2015; Chang et al., 2018; Chen et al., 2019), their occurrence is now known to extend throughout Southeast Asia (Senapin et al., 2019; Nurliyana et al., 2020; Meemetta et al., 2020), affecting the industry as a whole. To better understand what is happening to the biology of barramundi in commercial farming conditions during one such disease outbreak, the presence of five putative pathogens was investigated using a comparative RNAseq and histological approach targeting multiple organs of affected and non-affected juveniles. This study for the first time identified a concurrent infection of SDDV and LCHV in all barramundi samples (including all those apparently healthy), while ruling out infection of *Megalocytivirus* ISKNV/RSIV and two bacteria, *T. maritimum* and Vh-SDMN, which were associated with scale drop and muscle necrosis disease events in Vietnam (Dong et al., 2017b). Although lower LCHV detection in all samples were indicative of an underlying herpes viral coinfection, LCHV presence, or loading was not associated to any of the histopathological findings, or to clinical disease expression (sick/healthy groups) in the PCA. In contrast, kidney and liver SDDV loads were strongly associated with the severity of histopathological alterations observed in several organs, and sick individuals. Clinical disease, severity of pathology observed in the kidney and spleen, and viral loads support the diagnosis of primary scale drop disease, whereby apparently healthy fish were in subclinical stages while sick fish were in advanced disease stages of SDD. Furthermore, this study unveiled 2,810 and 556 differentially expressed genes in the liver and muscle respectively of sick and healthy fish, and importantly, identified immune-related pathways and genes which where predominantly downregulated in sick juveniles, thus contributing to broaden our understanding of the effects of SDD in barramundi farmed under commercial culture conditions.

The severity of tissue inflammation and necrosis in spleen and kidney of sick fish may explain why the RNA extracted from these organs were too degraded for further transcriptomic analyses. Severe and extensive tissue necrosis during the later clinical phase of SDD progression is expected to cause disruption of cellular, tissue, and organ functions. RNA is highly susceptible to degradation by reactive oxygen species (ROS), and oxidative RNA damage has been recently found to be involved in the pathogenesis of several chronic degenerative diseases (Fimognari, 2015). Barramundi spleen was the most affected organ by SDDV (and where RNA was most degraded in sick fish), followed by the kidney, liver, and muscle. In addition, histological observations from apparently healthy, but subclinically infected fish indicated that spleens are likely the first organ to be compromised by SDDV. The spleen stores erythrocytes (red pulp) and lymphocytes (white pulp) (Noga, 2006). The importance of spleen in modulating barramundi immune response and the severe damage observed in this organ may somewhat explain the inability of barramundi to fight against SDD and high mortality rates observed in farmed animals. Vaccination trials in barramundi against *S. iniae* revealed that spleen (but not the kidney) responded transcriptomically at 25–29 h postchallenge to activate NF_*K*_-B, chemokine, and toll-like receptor genes, whereby vaccinated fish had increased survival and reduced pathogen shedding (Jiang et al., 2014). Occlusion of splenic arteries from chronic obliterative endarteritis and subsequent multifocal splenic necrosis from infarcts are further evidence of strong non-specific innate inflammatory response and failure to control the SDDV infection. Pathology observed suggests that SDD is a chronic viral disease that develops over time (potentially weeks), before presenting as clinically diseased fish. The severe necrotizing glomerulonephritis in sick fish may be directly as a result of prolonged inflammation because of release of chemokines, interleukins, and tumor necrosis factors (TNF).

In this study, the KEGG metabolic pathway was the most enriched and significant pathway with 208 DEGs. Insulin signaling and AGE-RAGE signaling pathway in diabetic complications (among 27 immune-relevant pathways) were identified based on DEGs in sick vs. healthy fish, suggesting a disease-induced endocrine and metabolic disorder. In barramundi subjected to various stressors (LPS, *V. harveyi*, high salinity and fasting), DEGs associated with metabolic pathways were also notably downregulated (Xia et al., 2013). It is expected that genes associated with cellular processes and/or homeostasis will be affected by severe tissue damage during the advance stages of disease. It is likely that SDDV and underlying LCHV infections contributed to depletion of energy stores in liver from disease, when fish are stressed and stop feeding. Cortisol is known to cause insulin resistance which disrupts glucose metabolism (Kamba et al., 2016), and stress-related cortisol spikes are well described in fish (Sadoul and Geffroy, 2019). Starvation stage can cause abnormal fat accumulation in liver, from a disorder of glucose metabolism and energy for processing fat (Rui, 2014). There was consistently abnormal accumulation of fat macrovesicles in the liver (lipidosis) in all sick fish, in which functional hepatic genes (e.g., insulin-like growth factor binding protein 1a; progestin and adipoQ receptor family member Vb; glutamate receptor, ionotropic, AMPA 1a; fatty acid-binding protein 10a; phospholipase A2-like; etc.) were differentially expressed. Dietary fat is processed in the livers of fish very similarly to that in mammals, *via* lipoprotein conjugation and subsequent storage in adipose tissues throughout the body (Yan et al., 2015). In fact, ctrp1, a C1q/TNF-related adipokine strongly implicated in pathogenesis of non-alcoholic fatty liver disease (NAFLD), a human chronic liver disease associated with several metabolic-related disorders including insulin resistance (diabetes) and inflammation (Shabani et al., 2017), was one of the most significantly downregulated genes both in the liver and in the muscle of sick animals.

Genes associated with the adaptive immune system, in particular those involved in immune effector process, such as T cells, B cells, and the major histocompatibility complex (MHC) were also predominantly downregulated in SDD barramundi. SDDV differs from other systemic iridoviral disease, where infected cells are filled with large numbers of virions in crystalline array (Gibson-Kueh et al., 2003). In contrast, few mature virions are observed in fish with SDD using transmission electron microscopy (TEM), suggesting that SDDV infection results in the continual release of mature viral particles (Gibson-Kueh et al., 2012). It is also likely that continual viral shedding results in a prolonged inflammatory host immune response that causes severe tissue damage but fails to overcome SDDV infections. This is consistent with the observed involvement of a number of cytokines (e.g., chemokines, interleukins, and TNFs) and toll-like receptors, predominantly upregulated in SDD fish, which suggest an ongoing strong response by the innate immune system of fish in advance stages of SDD. Cytokines such as interleukins stimulate T cells, and TNF activates macrophages (Uribe et al., 2011). Toll-like receptors recognize viral infections and trigger the release of cytokines and chemokines (Xagorari and Chlichlia, 2008), a general mechanism which has also been previously observed in transcriptomic studies of barramundi infected by other pathogens such as *V. harveyi* (Xia et al., 2013), *S. iniae* (Jiang et al., 2014), and NNV (Liu et al., 2016). In the giant grouper (*Epinephelus lanceolatus*) infected with *Vibrio alginolyticus*, upregulation of the tlr5 gene leading to cytokine regulation has been suggested to induce proinflammatory and/or chemotactic effects (Wang et al., 2016). Here, tlr5 and tlr13 were significantly upregulated in both liver (8- and 33-fold) and muscle (56- and 83-fold) of sick fish, respectively. Similarly to what was observed in our study, tlr5 was upregulated by over 150-fold in spleen of barramundi challenged with *S. iniae* but not in vaccinated challenged fish (Jiang et al., 2014). Moreover, TLR signaling pathway has been shown ubiquitously upregulated in the intestines of barramundi after exposure to LPS, *V. harveyi* challenge, high salinity, and fasting (Xia et al., 2013). Taken together, these studies suggest that there is a coordinated response among several organs upregulating the TLR signaling pathway in response to pathogens, whereby our study suggests that tlr5 and tlr13 are key markers in SDD barramundi.

Lectins such as ladderlectin, L-rhamnose-binding lectin, and collectins were among the most differentially expressed genes in the livers of SDD barramundi. Lectins were identified as part of the barramundi immune response to foreign antigens (LPS) as early as 35 days posthatch (Xia and Yue, 2010). Lectins are assumed to mediate pathogen recognition, cell adhesion, the activation of complement pathway, and facilitate pathogen clearance by phagocytosis, thus playing an important role in innate immunity and disease resistance in fish (Elumalai et al., 2019). While the phagosome was the most enriched immune-related pathway in the livers of SDD barramundi, there was an overall downregulation of C3 and C1q, key complement proinflammatory genes in sick fish. Differential regulation of complement genes was observed in barramundi intestine, whereby LPS challenge upregulated C3 and downregulated C1q; the latter also downregulated at 40 h post-*V. harveyi* challenge (Xia et al., 2013). Complement genes coordinate the communication between the innate and the adaptive immune system (Bergman, 2011). Their activity is tightly regulated to avoid immune dysregulation and tissue damage as a consequence of excessive expression and inflammation (Wasiak et al., 2017). In giant grouper, *V. alginolyticus* challenge had time-dependent effects on several genes related to the complement pathway, with expression levels of most genes (including C3 and C1q) peaking between 4 and 8 h postinfection and returning to basal (preinfection) levels at 48 h postinfection (Wang et al., 2014). It is hypothesized here that the observed downregulation of C1q and C3 genes in SDD barramundi might be due to the fact that organs had already reached advanced inflammatory stages thus precluding further activation, which is not the case of apparently healthy, but subclinically SDDV- and LCHV-coinfected fish. However, the observed upregulation of C5 and C6 genes in sick fish might indicate that complement genes may be differentially activated in different phases in the animal’s immune response and disease progression.

Like in humans and other animals, collagen plays an important role in strengthening skin and its elasticity in the fish. If downregulation of collagen gene clusters in both liver and muscle of sick fish would also occur in skin tissue (not assessed in this study), it might possibly explain the observed damage in the epidermis and reduction of scale adhesion, which results in obvious scale detachment in clinically sick fish (for which SDD is known for). Systemic iridoviruses of fish target fibroblasts in connective tissues surrounding blood vessels (Gibson-Kueh et al., 2003). The inflammation in skeletal muscles involved mainly the connective tissues and not muscles, with the occasional presence of viral inclusion bodies. This is consistent with the lower number of DEGs, lower Pearson *R*^2^ values, and less evident heatmap clustering of muscle samples when compared with liver tissues between clinically sick and healthy *L. calcarifer*. This is also consistent with tissue predilection of systemic iridoviruses, targeting fibroblasts of mesothelial origin in all organs (Gibson-Kueh et al., 2003). The inflammation in dermis of skin is centered on the fibroblastic connective tissues of tunica adventitia of blood vessels. The marked occlusion of blood vessels would further explain infarct of epidermis of skin and scale loss characteristic of SDD, and the multifocal infarcts in spleen (Gibson-Kueh et al., 2012).

Finally, it is important to note that the transcriptomic comparison (and DEGs) between the healthy and sick fish groups evaluated in this field outbreak would likely be different if the comparison were made between the sick group and an “uninfected control group.” Such comparison would likely reveal more immune genes be identified as differentially regulated between sick and uninfected groups. Further studies investigating transcriptome of uninfected fish under laboratory conditions may be required to clarify this uncertainty. However, the data provided in this study might be an indication that SDDV (and LCHV) is currently endemic in sea-caged farmed barramundi juveniles within this site, which should also be confirmed by follow-up epidemiological surveys. This is not unlikely because in recent years, all batches farmed in the area get ubiquitously affected with SDD mortalities during the juvenile stages, whereby some animals get clinically sick and succumb to the disease, whereas others with mild infection and which look “apparently healthy” survive. Therefore, also of importance, future studies should evaluate when animals become infected after stocking, with time-series sampling plan over the course of multiple outbreaks (e.g., prior, during, and after) to better understand transcriptomic responses over the progression of disease.

In conclusion, SDDV infection (and to a lesser but unknown extent of LCHV coinfection) resulted in upregulation of genes associated with innate immunity, downregulation of genes associated with adaptive immunity and homeostatic regulation of cellular and tissue function, and severe inflammatory response that resulted in destruction of spleen followed by the kidney. Research to understand why SDD viral infection is not brought under control by the host immunity may be key to developing effective vaccines and/or immunostimulants to alleviate the effects of SDD in farmed barramundi.

## Data Availability Statement

The datasets presented in this study can be found in online repositories. The names of the repository/repositories and accession number(s) can be found below: NCBI SRA (Accession: PRJNA713978).”

## Ethics Statement

The animal study was reviewed and approved by the James Cook University Singapore, Animal Ethics Committee (Approval No. 2019-A07).

## Author Contributions

JD and DJ contributed to conception and design of the study. CT and MT collected specimens and organized the database. SS and HD performed the molecular diagnostics. XS and JD performed the RNAseq statistical analysis. SG-K performed the histopathology analysis. JD wrote the first draft of the manuscript. All authors contributed to manuscript writing, revision, read, and approved the submitted version.

## Conflict of Interest

The authors declare that the research was conducted in the absence of any commercial or financial relationships that could be construed as a potential conflict of interest.

## Footnotes

1 https://www.ncbi.nlm.nih.gov/genome/annotation_euk/Lates_calcarifer/100/

2 http://www.genome.jp/kegg/
