# Supplementary material for: Scale Drop Disease Virus (SDDV) and Lates calcarifer Herpes Virus (LCHV) Coinfection Downregulate Immune-Relevant Pathways and Cause Splenic and Kidney Necrosis in Barramundi Under Commercial Farming Conditions
Source: Front Genet. 2021 Jun 18;12:666897. doi: 10.3389/fgene.2021.666897 (PMC8249934; doi:10.3389/fgene.2021.666897)
Supplement: Supplementary file 2 [file Data_Sheet_2.docx]

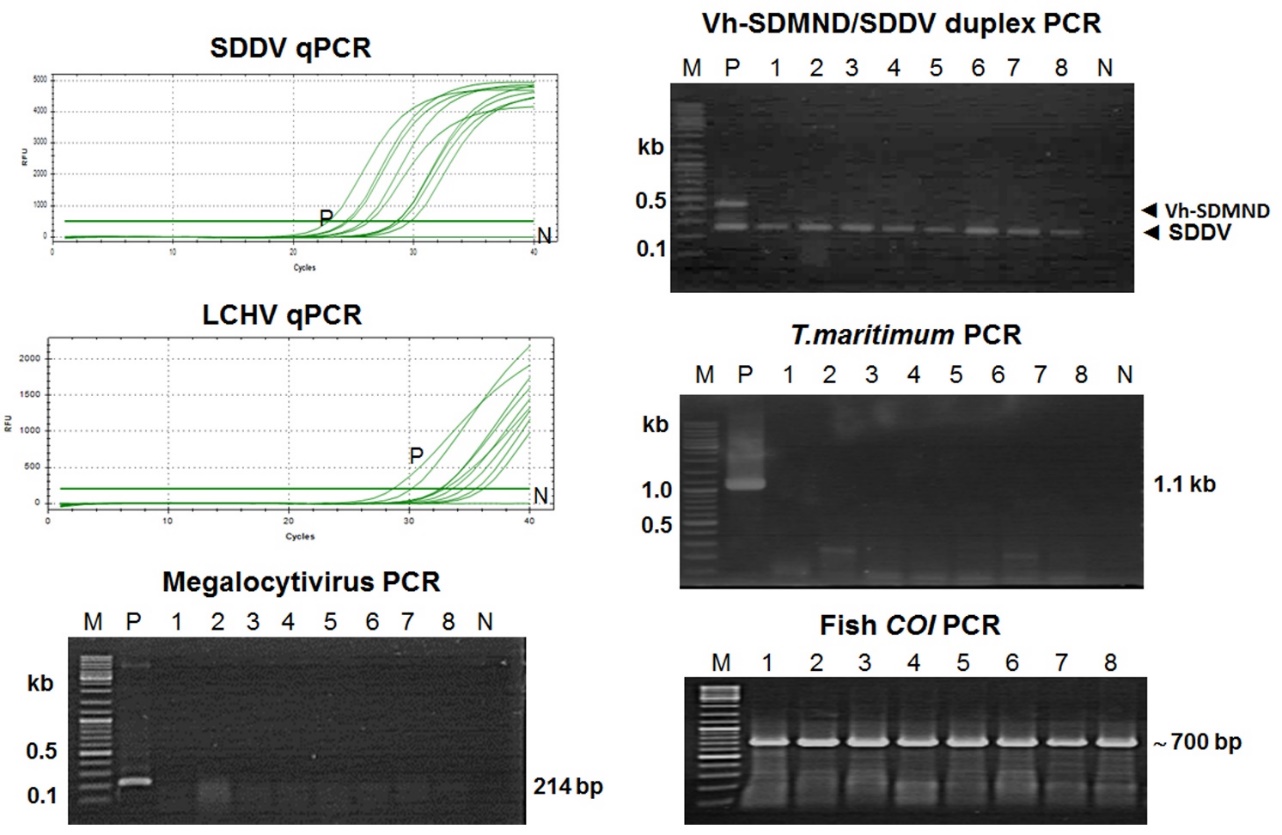


**Figure S1.** Representative qPCR and PCR test results of 8 DNA samples extracted from kidney of sick Asian sea bass. Pathogen screening was performed specifically for 3 viruses (SDDV, LCHV, Megalocytivirus) and 2 bacteria (Vh-SDMND and *T. maritimum*). Fish *COI* amplification was carried out to ensure amplifiable quality of DNA and comparable amount of the DNA template.


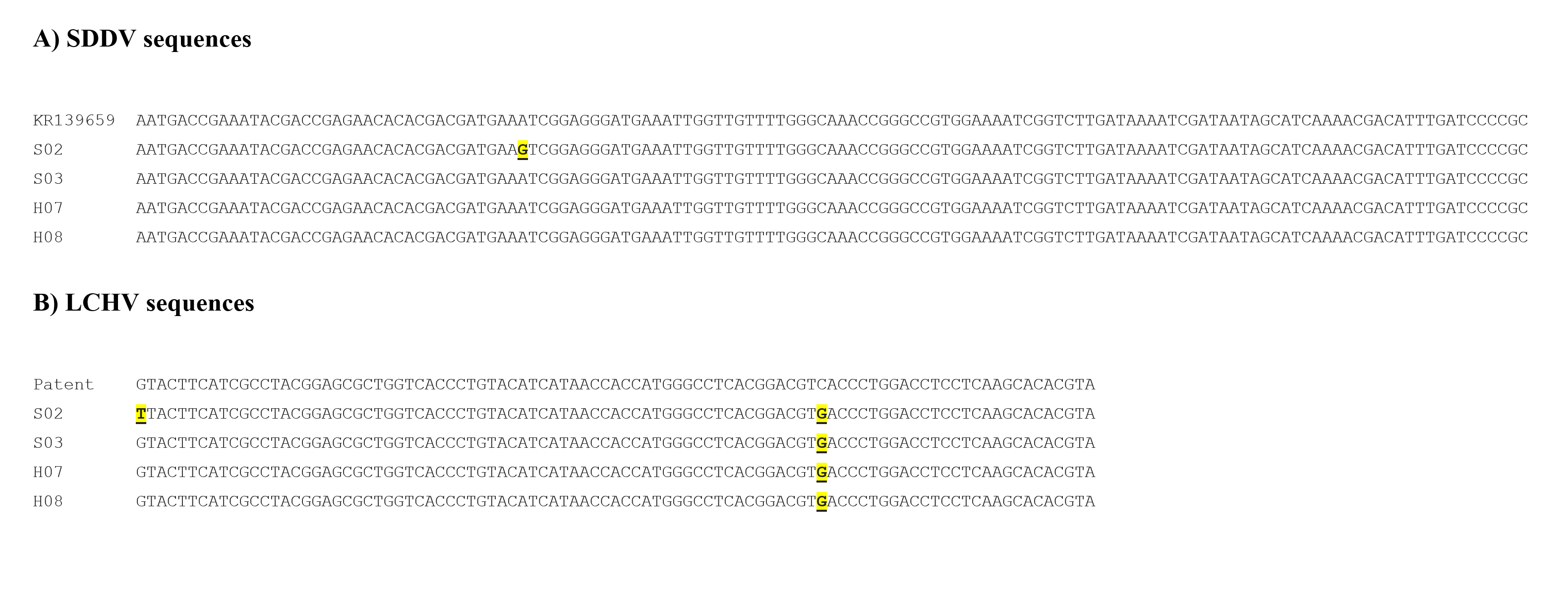
**Figure S2.** DNA sequence comparison of representative amplicons obtained from Sanger sequencing of qPCR product assays for detection of (A) SDDV and (B) LCHV. SDDV KR139659 is a sequence retrieved from GenBank, while LCHV Patent is a sequence retrieved obtained from internal patent number WO 2018/029301 AI.


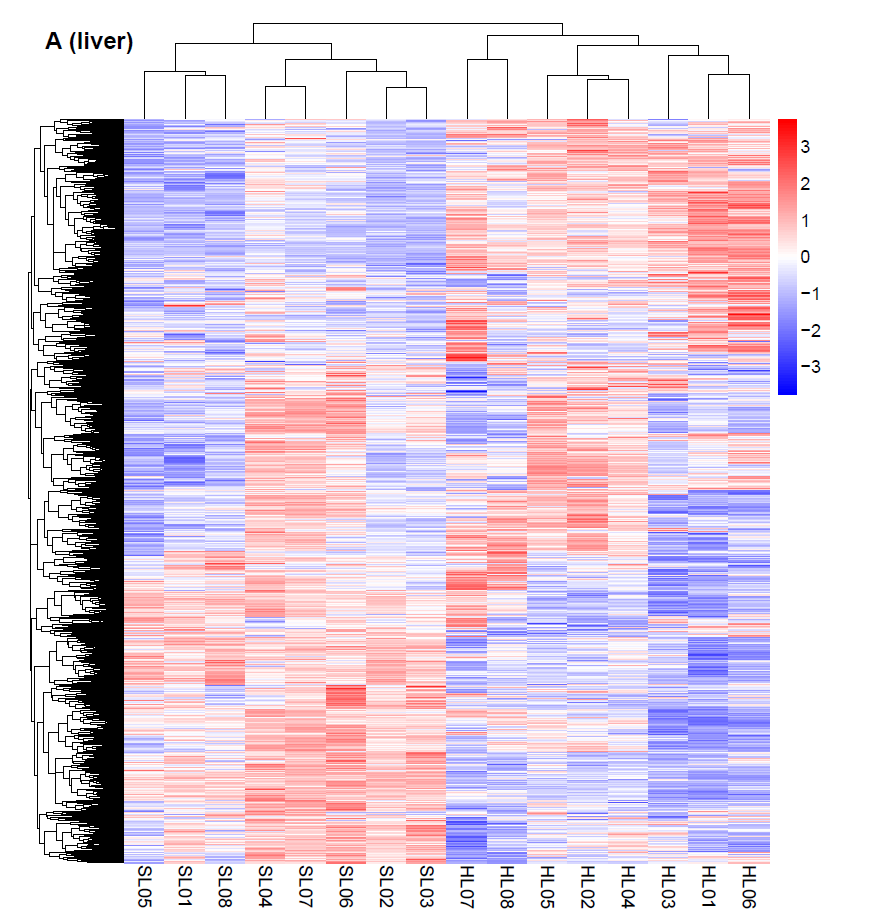

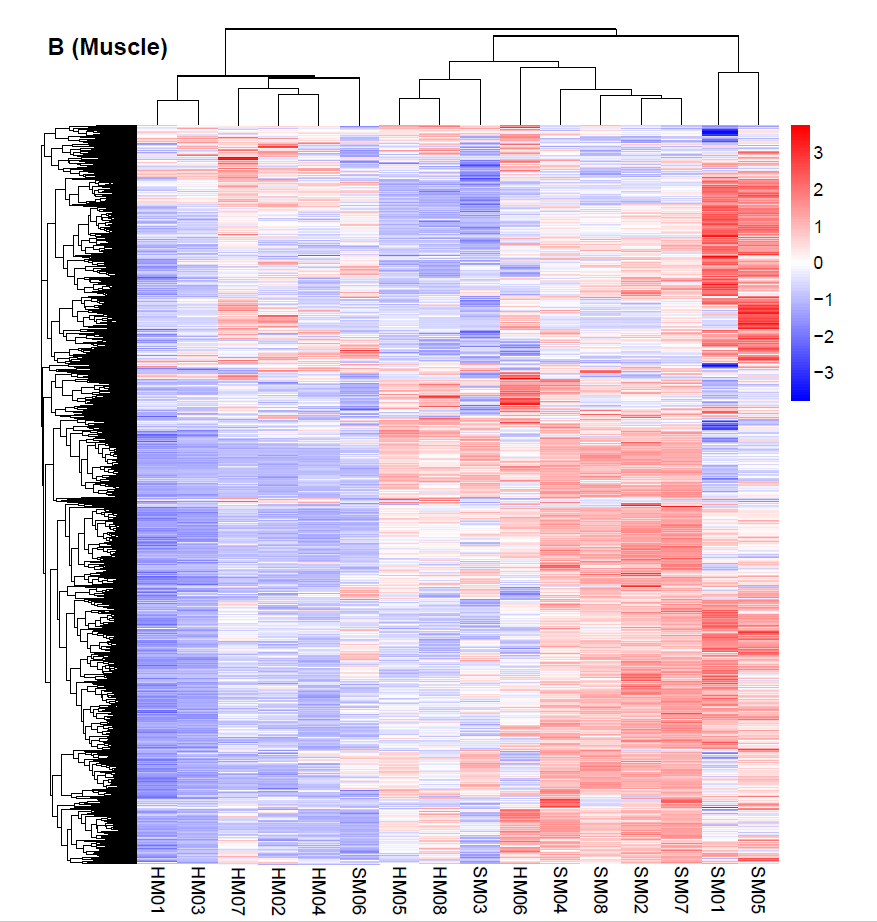


**Figure S3.** Heatmap analyses of the hierarchical clustering of DEGs in liver of sick (SL01 to SL08) and healthy (HL01 to HL08) fish (3A), as well as muscle of sick (SM01 to SM08) and healthy (HM01 to HM08) fish (3B). Each column representd an individual fish sample, and each row representd a gene. Intensity of blue and red colors in the heatmap correspond to low and high relative gene expression, respectively.


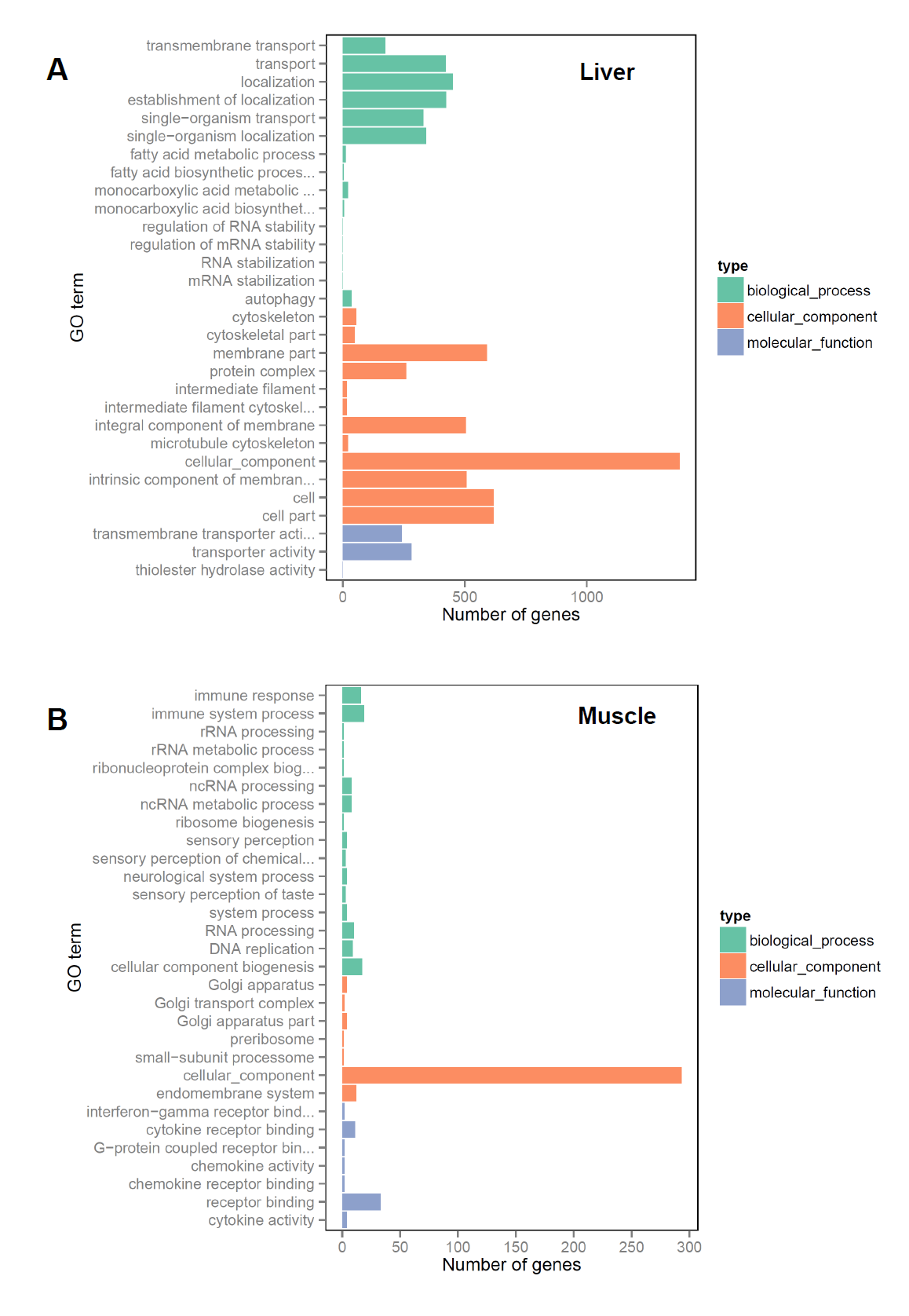


**Figure S4. A.** The most enriched GO terms in the liver and **B.** muscle of juvenile barramundi based on differentially expressed genes between sick and healthy animals.


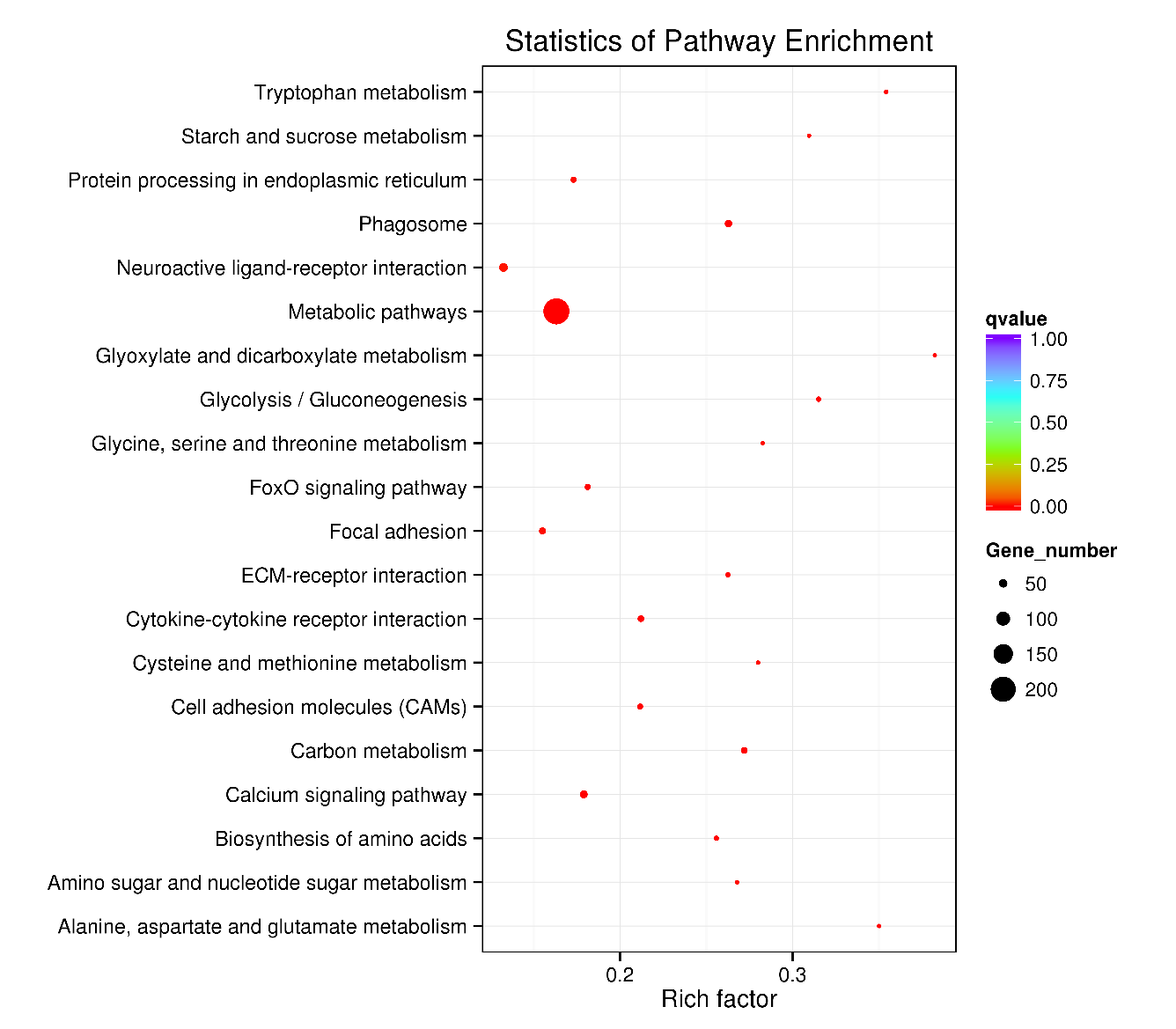


**Figure S5.** Scatterplot of the top 20 enriched KEGG pathways in liver. Rich Factor is the ratio of differential expressed gene numbers annotated in this pathway terms to all gene numbers annotated in this pathway term. Adjusted p value ≤ 0.05 as significantly enriched.


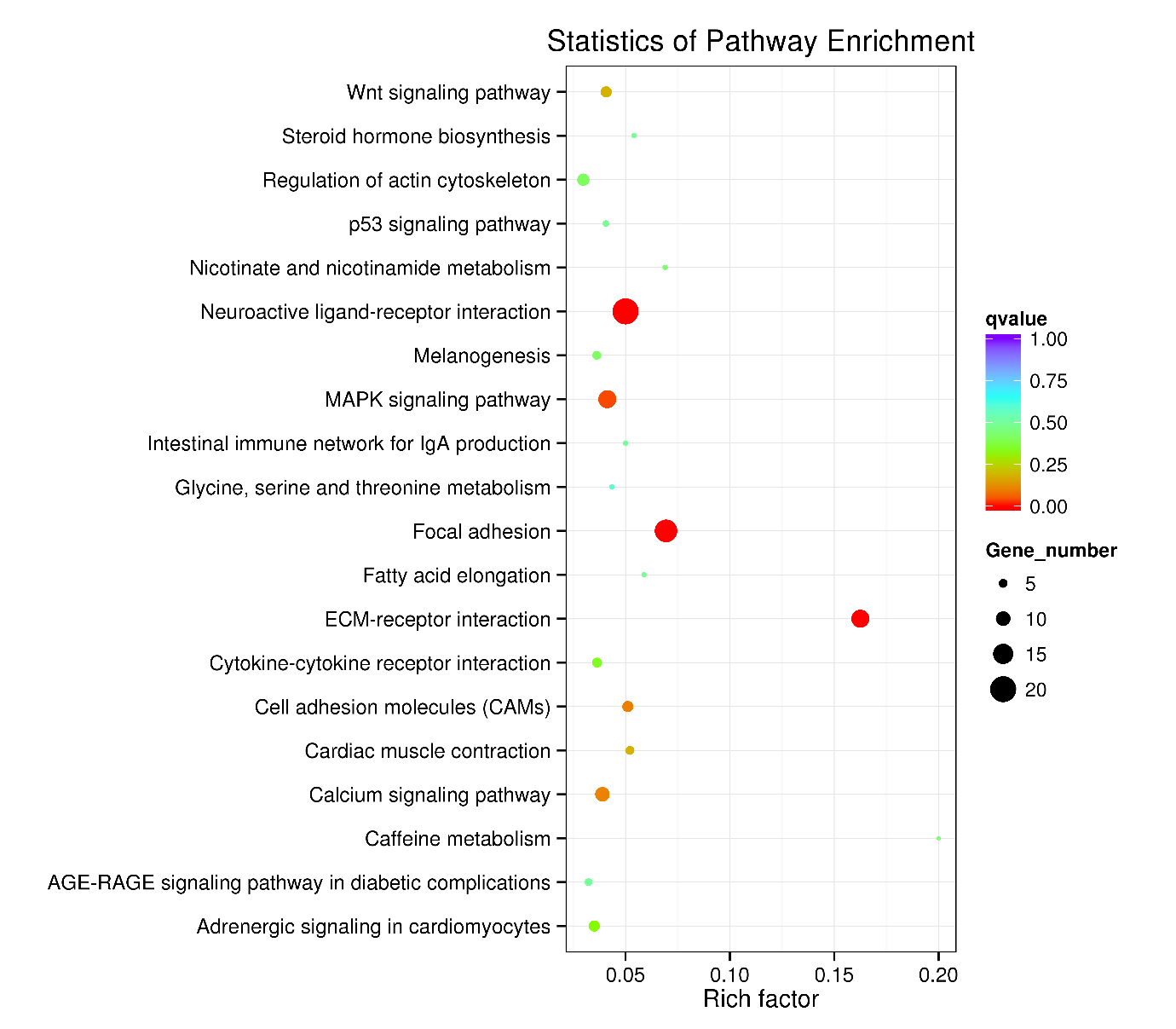
**Figure S6.** Scatterplot of the top 20 enriched KEGG pathways in muscle. Rich Factor is the ratio of differential expressed gene numbers annotated in this pathway terms to all gene numbers annotated in this pathway term. Adjusted p value ≤ 0.05 as significantly enriched.
